# Supplementary material for: Training deep learning models on personalized genomic sequences improves variant effect prediction
Source: bioRxiv. 2025 Feb 15:2024.10.15.618510. Originally published 2024 Oct 18. Preprint. [Version 2] doi: 10.1101/2024.10.15.618510 (PMC11507713; doi:10.1101/2024.10.15.618510)
Supplement: Supplement 1 [file NIHPP2024.10.15.618510v2-supplement-1.pdf]

bioRxiv preprint doi: https://doi.org/10.1101/2024.10.15.618510; this version posted February 15, 2025. The copyright holder for this preprint (which was not certified by peer review) is the author/funder, who has granted bioRxiv a license to display the preprint in perpetuity. It is made available under aCC-BY 4.0 International license.

349 ## Supplementary Figures

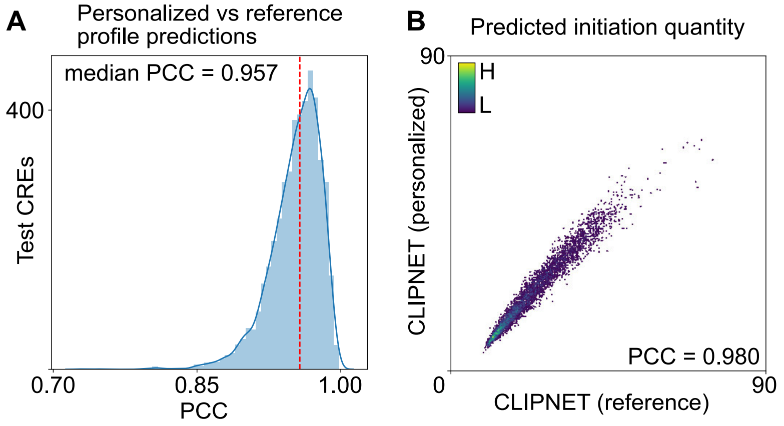

Figure S1: **Comparison of reference-trained and personalized CLIPNET predictions in LCLs across genomic loci.** (**A**) Initiation profiles predictions between reference-trained and personalized CLIPNET models are highly correlated (median profile Pearson's correlation between predictions = 0.957). (**B**) Initiation quantity predictions between reference-trained and personalized CLIPNET models are highly correlated (Pearson's correlation = 0.980). Points are colored by a Gaussian kernel density estimate.

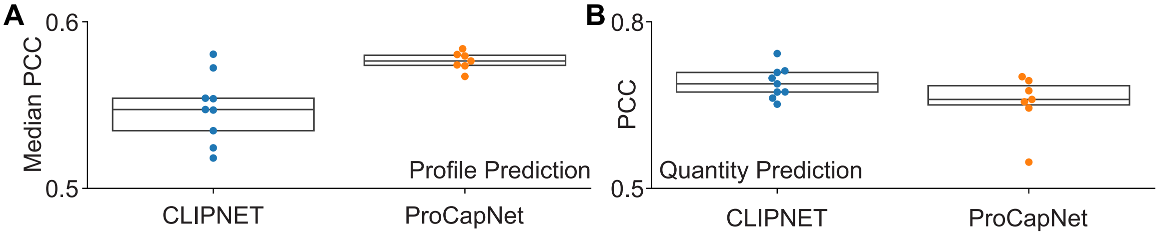

Figure S2: **Comparison of CLIPNET and ProCapNet cross-loci predictions in K562.** (**A**) Initiation profile prediction accuracy (median Pearson's correlation of model replicates). (**B**) Initiation quantity prediction (Pearson's correlation across model replicates). CLIPNET (n = 9) and ProCapNet (n = 7) models were evaluated on their respective holdout chromosomes. Box plots represent median and upper/lower quartiles.
