# Supplementary material for: Training deep learning models on personalized genomic sequences improves variant effect prediction
Source: bioRxiv. 2024 Oct 18:2024.10.15.618510. Preprint. [Version 1] doi: 10.1101/2024.10.15.618510 (PMC11507713; doi:10.1101/2024.10.15.618510)
Supplement: Supplementary file 1 [file NIHPP2024.10.15.618510v1-supplement-1.pdf]

bioRxiv preprint doi: https://doi.org/10.1101/2024.10.15.618510; this version posted October 18, 2024. The copyright holder for this preprint (which was not certified by peer review) is the author/funder, who has granted bioRxiv a license to display the preprint in perpetuity. It is made available under aCC-BY 4.0 International license.

Supplementary Figures

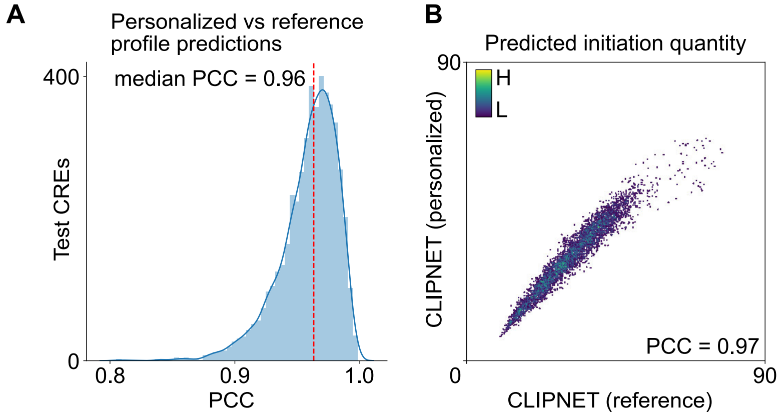

Figure S1: **Comparison of reference-trained and personalized CLIPNET predictions.** (**A**) Initiation profile prediction. (**B**) Initiation quantity prediction.

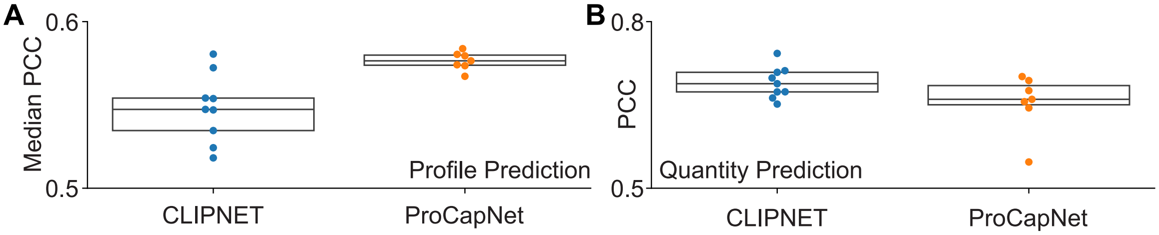

Figure S2: **Comparison of CLIPNET and ProCapNet cross-loci predictions in K562.** (**A**) Initiation profile prediction. (**B**) Initiation quantity prediction. CLIPNET (n = 9) and ProCapNet (n = 7) models were evaluated on their respective holdout chromosomes.
